# Do personality traits influence the stigmatizing attitudes toward people with mental illness? A web-survey among university students

**DOI:** 10.1192/j.eurpsy.2021.383

**Published:** 2021-08-13

**Authors:** L. Fusar-Poli, G. Saitta, M. Mormino, C. Avanzato, M.S. Signorelli, E. Aguglia

**Affiliations:** Department Of Clinical And Experimental Medicine, University of Catania, Catania, Italy

**Keywords:** personality traits, Stigma, University Students, big five inventory

## Abstract

**Introduction:**

People from the general population often tend to believe that psychiatric patients may be incurable, dangerous, and unpredictable. Stigma represents a critical issue which should be defeated. In spite of the interest of research, little is known about the relationship between personality traits and level of stigma toward people with mental illness.

**Objectives:**

To evaluate whether certain personality traits can influence the level of stigma towards mental illness in a population of university students.

**Methods:**

A web-survey was spread on social networks between March and June 2020 through Google Forms. Eligibility criteria for inclusion were:1) Being 18 years of age or older; 2) Attending a degree course in an Italian University; 3) Provide informed consent. Socio-demographic characteristics of the participants were collected. Stigma was measured using the Attribution Questionnaire (AQ-27), personality traits were evaluated through the Big Five Inventory (BFI) and the Mental Health Knowledge Schedule (MAKS-i) investigated the knowledge about mental illness. Statistical analyses were performed using SPSS 24.0.

**Results:**

We computed a multiple linear regression to calculate potential predictors of stigma, adjusted on the basis of the knowledge of mental illness. Results showed that age and faculty class were not related to stigma. Agreeableness (A) and Openness to experience (O) were associated with less stigmatizing attitudes. Conversely, Neuroticism (N) and Conscientiousness (C) seemed to predict higher levels of stigma.

**Conclusions:**

Our results suggest an interesting relationship between personality traits and stigmatizing attitudes, which deserves to be further studied. They also confirm the importance of implementing appropriate strategies against the stigma of mental illness.

**Disclosure:**

No significant relationships.

